# Defeating cancer with antidepressants

**DOI:** 10.3332/ecancer.2008.88

**Published:** 2008-08-21

**Authors:** J Lieb

**Affiliations:** 127 Cumberland Road, Burlington, Vermont 05408, USA 802 658 4909

## Abstract

Prostaglandins are ephemeral, infinitesimal signallers self-regulating every cell in the body, including those sub-serving mood and immunity. At first, they were perceived as a master switch, but now are believed to regulate every component of cellular micro-anatomy and physiology, including those of the organelles, cytoskeleton, proteins, enzymes, nucleic acids and mitochondria. Prostaglandins are responsible, paradoxically, for cell function and dysfunction. Excessive prostaglandin synthesis depresses immune function and may induce cancer. An ideal anti-cancer agent would inhibit prostaglandins in such a manner as to shut down the pathogenesis of cancer. In this paper, I will show that antidepressants have such properties.

## The anti-prostaglandin, immunostimulating and antimicrobial properties of lithium and antidepressants

Depression predisposes us to, among other things, infection, cancer, osteoporosis, and neurodegenerative, cardiovascular and auto-immune disorders [1,2]. Excessive syntheses of prostaglandins is incriminated in all of these [1,2]. Lithium and antidepressants have potent anti-prostaglandin, immunostimulating and antimicrobial properties, and antidepressants have the paradoxical ability to mitigate, reverse or induce auto-immunity [1,2].

When synthesized excessively, prostaglandin E2 depresses cellular and humoral immunity, allowing pathogens to replicate 3. Prostaglandins regulate the physiology, immunity, replication and toxicity of micro-organisms and the resistance of their hosts [1–4]. Failure of non-steroidal anti-inflammatory drugs in infections led to the conclusion that inhibiting prostaglandins has limited value in that field. The prostaglandin-inhibiting properties of lithium and antidepressants have been neglected [5–10], along with their unique immunopotentiating and antimicrobial actions [2]. In the early 1950s, clinicians observed that patients treated for tuberculosis with the monoamine oxidase inhibitors isoniazid and iproniazid had an elevation of mood and energy. It was also observed that monoamine oxidase inhibitors have dual anti-tuberculosis and antidepressant properties failed to impact the pharmacology of infectious disorders. Remission of such manifestations of viral infections as sinusitis, sinobronchitis, frequent colds, sore throats, cold sores and genital herpes in patients taking lithium carbonate has been reported [11–13]. The polymorphonuclear leukocytes of a 29-year-old woman with eczema and recurrent staphylococcal and streptococcal skin infections were unresponsive to standard chemotactic stimuli. *In vitro* addition of lithium to her polymorphonuclear preparations restored their chemotactic response. After receiving lithium carbonate, 1 g/d for five weeks, she became free of infection and relapsed when lithium was withdrawn [14]. Lithium chloride prevents replication of type 1 and 2 herpes virus in cell culture [15] and augments several *in vitro* immune reactions [16].

Monoamine oxidase inhibitors can reverse tuberculosis, aphthous ulcers, cold sores, genital herpes, upper respiratory tract infections and plantar warts [17–19]. Tricyclic antidepressants can reverse aphthous ulcers [20], reduce the frequency of recurrences of shingles [1,2], remit the pain of this disorder [1,2], prevent post-herpetic neuralgia [1,2], destroy leishmania minor and major *in vitro* [21], and inhibit *in vitro* growth of the intestinal parasite giardia lamblia [22]. Tricyclic antidepressants have anti-malarial properties: they enhance *in vitro* susceptibility of *Plasmodium falciparum* to chloroquine and are lethal *in vitro* against *Trypanasoma* parasites [23–27]. Selective serotonin re-uptake inhibitors can destroy such fungi *in vitro* as *Candida* and *Aspergillus* [28], reverse recurrent vulvovaginal candidiasis *in vivo* [29], have anti-microbial activity [30] and are synergistic when combined with antibiotics [31].

Impaired lymphocyte function reduced natural killer cell activity, reduced lymphocyte responses to mitogens and decreased natural killer cell populations have been demonstrated in depressives [1,2,32,33]. Tricyclic antidepressants augment natural killer cell activity *in vivo* and *in vitro* [34] and the monoamine oxidase inhibitor tranylcypromine enhances defective cell-mediated immunity [35]. As lithium and antidepressants have immunopotentiating properties, they are effective against a wide range of micro-organisms. Evidence to date shows that while lithium has antiviral and antibacterial properties, antidepressants have antiviral, antibacterial, antiparasitic and fungicidal properties. Response of infection to lithium and antidepressants mirrors that of response to depression, with subjects responding selectively to antidepressants or lithium; antidepressants are highly specific and humans remarkably variable. Response of depression and infection to lithium or an antidepressant is usually simultaneous, suggesting that the central actions of the drugs are important. While antivirals are not necessarily immunostimulants, lithium and antidepressants are invariably antivirals. If antidepressants double as antibiotics, it would not be surprising if antibiotics doubled as antidepressants. Many antibiotics, among them clarithromycin, erythromycin, amoxacillin and ciprofloxacin, can elevate mood to the level of hypomania or mania [36].

## Prostglandins in carcinogenesis

Among the mechanisms of carcinogenesis are up-regulation of cyclo-oxygenase, oncogene synthesis and expression, viral activation, signal disruption, accelerated cell replication, failed apoptosis, tumour initiation and promotion, angiogenesis, metastasis, immunosuppression, auto-immunity and activation of mitochondria. All fall within the orbit of prostaglandins and their forming enzymes. In 1968, Williams reported high levels of prostaglandins in the thyroid and plasma of patients with medullary cancer of the thyroid [37]. In 1976, Goodwin reported excessive synthesis of prostaglandin E2 in suppressor T-cells of patients with Hodgkin’s disease [38]. Numerous studies have confirmed elevated levels of prostaglandins in solid tumours and in the immune cells and body fluids of cancer patients [39,40]. The isolation of such isoforms of cyclo-oxygenase as COX-2 [41], and the synthesis of selective COX-2 inhibitors has stimulated research into the expression of this isoform in cancer and its role in apoptosis. COX-2 is up-regulated in such cancers as those of the head and neck, breast, lung, pancreas, bladder, cervix, prostate and mesothelium [41–43]. In population studies, chronic use of such prostaglandin inhibitors as aspirin and ibuprofen has reduced the risk of colon cancer by as much as 40% [44].

Armato and Andreis showed that arachidonic acid and prostaglandins F1 alpha and F2 alpha stimulate the DNA-synthetic and mitotic activities of hepatocytes [45]. Goodlad has reported that the increase in gastric mucosal mass induced by misoprostol in the stomach of dogs is due to increased cell production. The increase in mucosal mass was the result of a dramatic increase in the foveolar surface mucous cells [46], other studies show a paradoxical, inhibitory effect of prostaglandins on DNA synthesis [47]. Prostaglandins and their synthesizing enzymes are key factors in many signalling events, and disruptions of signalling pathways have been incriminated in many cancers.

In her pioneering studies, Karmali [48,49] showed that increased thromboxane formation in human breast cancer specimens is associated with three clinical variables: tumour size, axillary lymph node metastases and distant metastases. The mechanisms by which prostaglandins and thromboxanes induce metastasis include induction of proteolytic enzyme production, neovascularization and subversion of the immune response. The initiation of metastasis is thought to involve the adherence of circulating tumour cells to endothelial cells or to basement membranes. Prostaglandins and thromboxanes play a role in adherence [49,50], with local thromboxane concentrations possibly determining the sites of metastasis [51]. Immunosuppression is a cause and effect of cancer. Increase in prostaglandins at the primary tumour focus may block surveillance by the immune system, while an increase in plasma prostaglandins may contribute to the suppressive environment for lymphocyte function [52].

In a paradoxical counterpoint to immunosuppression, numerous autoimmune phenomena are reported in patients with cancer [53]. Malignant tumours are diagnosed with increased frequency in patients with such autoimmune disorders as pemphigus, Myasthenia gravis and the Eaton-Lambert syndrome [54,55]. The paraneoplastic syndrome includes a variety of neurological, haematological, metabolic, cardiovascular and dermatological disorders, in all of which prostaglandins have been incriminated [55,56]. As monoamine oxidase inhibitors, originally used in the treatment of tuberculosis, have potent antiviral and immunostimulating properties, it is not surprising that one of them, Matulane (procarbazine), is effective in treating stage 111 and 1V Hodgkin’s disease.

## Depression: A precursor of cancer

In the Ward Jones lecture given at Manchester University in 1957, Sir Heneage Ogilvie commented, ‘I have slowly come to frame in my mind an aphorism that can never be stated as such, because no statistics can be advanced to support it: “The happy man never gets cancer” … The instances where the first recognisable onset of cancer has followed almost immediately on some disaster, bereavement, the breakup of a relationship, a financial crisis, or an accident are so numerous that they suggest that some controlling force that has hitherto kept the outbreak … in check has been removed’ [57]. In 1998, Penninx *et al* at the National Institute of Aging provided compelling data for Ogilvie’s hypothesis: chronically depressed people over the age of 70 are 88% more likely to develop cancer and twice as likely to die of it than their mellow peers [58].

## Antineoplastic properties of antidepressants *in vitro*

Many studies show that antidepressants have potent anti-cancer properties, both *in vitro* and *in vivo*, with regard to various antidepressants, mechanisms of action and cancer cell types [59–78]. Irrespective of their putative mechanism of action, the antidepressants destroyed the cells or arrested their proliferation [59–78]. Hydroxyprostaglandin dehydrogenase is the primary prostaglandin-degrading enzyme, highly expressed in normal colon mucosa but lost in human colon cancers [79,80]. Lack of this enzyme promotes the earliest steps of growth of benign as well as malignant colon tumours [79,80]. When this enzyme was first characterized, every agent tested in the hope of stimulating it either had no effect or inhibited it. Eventually Mak and Chen showed that amitriptyline and imipramine powerfully activate the enzyme in mice, especially the kidney enzyme, with more than a thousand-fold activation by amitriptyline. Amitriptyline and imipramine had potent activating effects on this enzyme in the brain [81].

## Mitochondria, prostaglandins and antidepressants

Mitochondria are tiny organelles that supply cellular energy and are involved in signalling, cellular differentiation, control of the cell cycle, growth and programmed cell death. The cells of malignant gliomas of the brain, and small and non-small cell cancers of the lung, tend to repair DNA-breaks caused by radiation and chemotherapy. In an effort to accomplish cell death by an alternative method, investigators are targeting mitochondria. Small molecule agents known as ‘mitocans’ are able to enter tumour cell mitochondria, reduce oxygen consumption, and activate mechanisms leading to cell death. Agents that can destroy cancer cells in this manner, while leaving normal cells intact, notably include antidepressants [82–84]. Laboratory experiments using this approach on various cancer cells, including those of gliomas, are encouraging [65,66]. It goes without saying that prostaglandins are intermediaries between mitocans and mitochondria [85–87].

## Antineoplastic properties of antidepressants *in vivo*

A woman suffering from major depression and advanced liver cancer (hepatoma) was treated with psychotherapy, the antidepressant fluvoxamine (Luvox), glycyrrhizin acid and dehydroepiandrosterone (DHEA). Various indices of defective immune function normalized, and her liver function tests improved. At follow-up two-and-a-half years later, she was well and symptom free [88]. In 1990, a 60-year-old woman had a mastectomy for inflammatory breast cancer, followed by excision of infiltration of the chest wall. She was given a prognosis of less than a year. I treated her with various antidepressants, and when relocating in 2003 she was in apparent good health.

A middle-aged man had a two-year history of recurrent glioblastoma multiforme of the cerebellum, resistant to all conventional therapies. Within a week of starting sertraline, he noticed an appreciable reduction in tremor and ataxia. After taking the antidepressant for three weeks, he was virtually free of these symptoms.

## Discussion

It would seem that antidepressants have the potential to arrest, prevent, reverse and palliate cancer. Short of that they have many other uses in cancer care. Antidepressants can reduce the severity and frequency of hot flashes in patients treated with chemotherapy for breast cancer, and venlafaxine (Effexor), and remit acute neurosensory symptoms secondary to oxaliplatin chemotherapy [89]. The monoamine oxidase inhibitors deprenyl and clorgyline protect nonmalignant human cells from ionising radiation and chemotherapy toxicity [90], and such antidepressants as nefadazone are capable of reversing chemotherapy-induced vomiting [91].

As the response to antidepressants is highly specific, many patients require multiple trials before responding. Some subjects are refractory to all antidepressants, and some relapse due to tachyphylaxis [92]. Prostaglandins are capable of paradoxically inducing pro- and anti-cancer actions. The omnipresence of paradox warns that antidepressants are capable of initiating or accelerating cancer. Maintaining an index of suspicion, close clinical observation and limiting the duration of drug trials can mitigate such paradox. Epidemiological studies have failed to confirm the suspicion that antidepressants may induce breast cancer [93]. However, breast cancer has been reported in three men, taking selective serotonin re-uptake inhibitors [94].

Wherever prostaglandin-synthesizing enzymes convert arachidonic acid or phospholipids to prostaglandins, there are possible sites of action of antidepressants. By maintaining these enzymes within physiological limits, antidepressants shut down the mechanisms of carcinogenesis. Considerable evidence now shows that antidepressants are cytotoxic and cytostatic; convert multidrug resistant cells to sensitive and protect nonmalignant cells from ionizing radiation and chemotherapy [95]. While lithium has immunostimulating and anti-microbial properties, there is little evidence for its possible antineoplastic actions. Antidepressants have potent analgesic properties alone or as potentiators of narcotics, and they enhance sleep, appetite and occasionally energy. Their immunostimulating and anti-microbial properties are relevant to infection secondary to chemotherapy or radiation. Alleviation of anxiety, depression, recrimination and remorse by antidepressants can be very beneficial.
